# Application of the Capability, Opportunity, Motivation and Behavior (COM-B) model to identify predictors of two self-reported hand hygiene behaviors (handwashing and hand sanitizer use) to prevent COVID-19 infection among U.S. adults, Fall 2020

**DOI:** 10.1186/s12889-022-14809-y

**Published:** 2022-12-16

**Authors:** Laura G. Brown, E. Rickamer Hoover, Bethlehem N. Besrat, Claire Burns-Lynch, Rebekah Frankson, Shantrice L. Jones, Amanda G. Garcia-Williams

**Affiliations:** 1 National Center for Environmental Health, U.S. Centers of Disease Control and Prevention, 4770 Buford Highway, Atlanta, GA 30341 USA; 2 Division of Foodborne, Waterborne, and Environmental Diseases, U.S. Centers of Disease Control and Prevention, Atlanta, GA 30341 USA

**Keywords:** Handwashing, Hand sanitizer, Hand hygiene, COM-B model, Behavioral theory

## Abstract

**Background:**

Handwashing with soap and water is an important way to prevent transmission of viruses and bacteria and worldwide it is estimated handwashing can prevent 1 in 5 viral respiratory infections. Frequent handwashing is associated with a decreased risk for infection with SARS-CoV-2, the virus that causes coronavirus disease 2019 (COVID-19). Using a hand sanitizer with at least 60% alcohol when handwashing is not feasible can also help prevent the transmission of viruses and bacteria.

**Objective:**

Since early 2020, the public has been encouraged to handwash frequently with soap and water and use alcohol-based hand sanitizer when soap and water are not available to reduce COVID-19 transmission. This study’s objectives were to assess U.S. adults’ perceptions of components of the Capability, Opportunity, Motivation and Behavior (COM-B) Model in relation to these two hand hygiene behaviors and to identify relationships between these components and hand hygiene behaviors.

**Methods:**

Items assessing capability, opportunity, motivation, and hand hygiene behaviors were included in *FallStyles,* a survey completed by 3,625 adults in the fall of 2020 through an online panel representative of the U.S. population. We calculated composite capability, opportunity, and motivation measures and descriptive statistics for all measures. Finally, we conducted multiple logistic regressions to identify predictors of handwashing and hand sanitizer use.

**Results:**

Most respondents reported frequently washing hands with soap and water (89%) and using alcohol-based hand sanitizer (72%) to prevent coronavirus. For capability, over 90% of respondents said that neither behavior takes a lot of effort, but fewer agreed that they knew when, or how, they should engage in handwashing (67%; 74%) and hand sanitizer use (62%; 64%). For opportunity, over 95% of respondents said lack of time didn’t make it hard to engage in either behavior; fewer said visual cues reminded them to engage in the behaviors (handwashing: 30%; sanitizer use: 48%). For motivation, the majority believed the two behaviors were good ways to prevent coronavirus illness (handwashing: 76%; sanitizer use: 59%). Regressions indicated that capability, opportunity, and particularly motivation were positively associated with both hand hygiene behaviors.

**Conclusions:**

The COM-B model was a helpful framework for increasing understanding of hand hygiene behavior; it identified capability, opportunity, and motivation as predictors of both handwashing and hand sanitizer use.

## Introduction

Handwashing with soap and water is an important way to prevent transmission of viruses and bacteria and worldwide it is estimated handwashing can prevent 1 in 3 diarrheal illnesses and 1 in 5 viral respiratory infections [1]. More recent research indicates that frequent handwashing is associated with a decreased risk for infection with SARS-CoV-2 [2], the virus that causes coronavirus disease 2019 (COVID-19), and which has led to 1,080,470 deaths in the U.S. since January 2020, as of December 2022 [3]. Using a hand sanitizer with at least 60% alcohol when handwashing is not feasible can also help prevent the transmission of viruses and bacteria; alcohol-based hand sanitizers can inactivate many types of microbes, including SARS-CoV-2 [4, 5].

Since the beginning of the COVID-19 pandemic, the U.S. Centers for Disease Control and Prevention (CDC) has included frequent handwashing with soap and water (and the use of alcohol-based hand sanitizer when handwashing is not feasible) in its recommendations to prevent the spread of SARS-CoV-2 [6]. Hand hygiene recommendations became less prominent after COVID-19 vaccines became available (December, 2020) and CDC indicated that inhalation of very fine respiratory droplets and aerosol particles was a primary source of SARS-CoV-2 transmission (May, 2021). However, CDC continues to recommend hand hygiene behaviors to protect against SARS-CoV-2 transmission [6]. Multiple studies conducted during the COVID-19 pandemic have described U.S. adults’ hand hygiene behaviors (i.e., handwashing and hand sanitizer use). Researchers have found moderate to high levels of engagement of handwashing and hand sanitizer use among U.S. adults [7–15]. However, certain populations have lower levels of self-reported hand hygiene behavior, including men and young adults [7, 10, 11, 16, 17].

Numerous studies have described factors associated with hand hygiene behavior during the COVID-19 pandemic. These include demographic factors such as gender, age, income, education, and self-rated health [7, 8, 10, 11, 16–19] and psychosocial factors like risk perceptions, perceptions of severity of COVID-19, previous experience with COVID-19, knowledge about COVID-19, trust in government, reflective motivation, capability and opportunity to engage in hygiene behaviors, self-efficacy, cues to action, perceived ease of engaging in the behavior, and positive outcome expectancies [7, 10, 13, 15, 17, 19–26].

An increasing number of studies have used behavioral science theories to guide formative research on hand hygiene behaviors during the COVID-19 pandemic [13, 19, 22, 24, 27, 28]. Behavioral science theories provide a framework with which to understand why individuals engage or do not engage in a range of positive or negative health behaviors [29], and it is recommended that theory be used to guide the development of health communication materials and behavior change interventions in general [30], and during infectious disease outbreaks [31]. Theories used have included the Social Cognition Model, the Health Belief Model, the Reasoned Action Approach, the Multi-Theory Model of Health Behavior, the Integrated Behavioural Model for Water, Sanitation, and Hygiene, and the Capability, Opportunity, Motivation, and Behavior (COM-B) model. To contribute to theory-driven efforts, the current study uses the COM-B Model to identify determinants of washing hands with soap and water and hand sanitizer use in a probability-based sample of U.S. adults. The COM-B model posits that for an individual to engage in a particular behavior, they must have the capability, opportunity, and motivation to do the behavior [32]. The objective of this study is to identify factors that influenced hand hygiene behaviors among U.S. adults in the fall of 2020 period of the COVID-19 pandemic so that these factors can be targeted through theory-driven communication, education, and behavior change interventions. This work can also inform broader efforts to improve hand hygiene behaviors among U.S. adults.

## Methods

In 2020, Porter Novelli Public Services conducted several surveys via Ipsos’ KnowledgePanel, an online panel that is representative of the non-institutionalized U.S. population [33]. Respondents are randomly recruited by mail through probability-based, stratified sampling to participate in the survey panel. Respondents that agree to participate receive the surveys through email and complete them online; they are provided internet access and a computer, if needed. Respondents receive reward points they can redeem online for gift cards or prizes. The fall wave of the survey, *FallStyles*, was conducted from September 24 – October 10, 2020 (prior to the availability of COVID-19 vaccines). The survey was sent to a sample of 4,548 panelists ages 18 or older who answered the spring wave (June, 2020) of the survey. Email reminders were sent to non-responders on day 3, 7 and 13 of the survey period. Respondents completed surveys in a median time of approximately 33 min. Respondents were not required to answer any of the questions and could exit the survey at any time; data from respondents who did not answer at least half of the questions or completed the survey in five minutes or less were removed from the dataset (*n* = 8). A total of 3,625 adults completed the survey for a response rate of 79.7%. The analyses were weighted to match the U.S. Census 2019 U.S. Current Population Survey proportions by gender, age, annual household income, race/ethnicity, household size, education, census region, metropolitan status, and parental status. See the Porter Novelli website for more information on *ConsumerStyles* methodology [33].

### Demographic variables

Data on multiple demographic characteristics were collected when respondents agreed to participate in the survey panel. Of those, gender, age, income, education, race/ethnicity, and geographic region (based on U.S. Census regions) were included in our analyses as statistical controls.

### Hand hygiene variables

To assess hand hygiene precautions taken in response to COVID-19, respondents were asked, “What, if any, precautions are you taking to prevent coronavirus?”. They were then provided a list of precautions, presented randomly, and asked to endorse those they engaged in. We present data on the following hygiene-related behaviors from the list of precautions: washing hands often with soap and water and using alcohol-based hand sanitizer (hereafter referred to as handwashing and hand sanitizer use) (Table 1).

**Table 1:** Demographic characteristics of respondents overall, and among respondents reporting washing their hands often and using alcohol-based hand sanitizer to prevent coronavirus – Fall Styles data, 2020 (Unweighted *N* = 3,625)

		**Overall**		**Handwashing**		**Hand Sanitizer**	
		N^a^	%^b^	N^a^	%^b^	N^a^	%^b^
**All respondents**	**-**	–	–	3,237	88.6	2,584	71.5
**Age**	18–29	324	19.2	272	85.6	223	69.0
	30–44	765	26.0	656	84.9	552	71.9
	45–59	950	25.6	849	89.6	683	72.0
	≥ 60	1,583	29.2	1,460	93.0	1,126	72.5
**Gender**	Female	1,772	51.6	1,606	91.2	1,332	75.4
	Male	1,850	48.4	1,631	85.9	1,252	67.4
**Race/Ethnicity**	Non-Hispanic White	2,709	63.9	2,403	87.8	1,885	69.3
	Non-Hispanic Black	280	11.4	261	91.2	211	75.0
	Hispanic	339	16.1	307	89.5	254	73.6
	Multiracial/other	294	8.6	266	89.7	234	80.0
**Education**	< High school	194	10.8	162	82.4	129	67.2
	High school	994	28.2	878	89.0	690	69.9
	Some college	969	27.8	865	88.6	691	71.3
	≥ Bachelor’s degree	1,465	33.8	1,332	90.1	1,074	74.4
**Income**	< $25 k	399	13.4	335	82.7	262	67.0
	$25 k–$49 k	623	17.8	564	90.5	426	69.8
	$50 k–$99 k	1,099	30.7	981	88.2	789	72.2
	≥ $100 k	1,501	38.1	1,357	90.2	1,107	73.4
**Region**^c^	Northeast	646	17.6	594	90.8	464	71.5
	Midwest	826	20.7	720	86.7	578	70.7
	South	1,249	37.8	1,128	89.7	911	73.3
	West	901	24.0	795	86.9	631	69.5

^a^unweighted N

^b^weighted %

^c^Region categories based on U.S. Census

### COM-B variables

To assess the COM-B constructs of capability, opportunity and motivation to engage in hand hygiene behaviors to prevent coronavirus infection, respondents were presented with a series of statements about each hand hygiene behavior and asked whether they agreed with the statements. Items were developed based on the definitions of the COM-B constructs, example hand hygiene items from *The Behaviour Change Wheel: A Guide to Designing Interventions* [34], and from the COM-B Hand Hygiene Behavior Questionnaire [35]. For handwashing and hand sanitizer use, respondents responded to six statements about capability (e.g., I am capable of frequently washing my hands/using hand sanitizer to prevent coronavirus), four statements about opportunity (e.g., Frequent handwashing/frequently using hand sanitizer is a habit in my everyday life), and four statements about motivation (e.g., Frequent handwashing/Frequent use of hand sanitizer is a good way to protect myself from coronavirus) (Table 2).

**Table 2:** Descriptive statistics for measures of respondents’ perceived capability, opportunity, and motivation to frequently hand wash and use hand sanitizer– Fall Styles data, 2020 (*N* = 3,625^a^)

	**Handwashing**				**Hand Sanitizer use**			
**Capability**	**N**^**a**^	**%**^**b**^	**–**^**c**^	**–**^**c**^	**N**^**a**^	**%**^**b**^	**–**^**c**^	**–**^**c**^
I am capable of frequently washing my hands/using hand sanitizer to prevent coronavirus^d^	2,843	75.7	**–**	**–**	2,447	65.4	**–**	**–**
I know when I should wash my hands/use hand sanitizer to prevent coronavirus^d^	2,495	67.0	**–**	**–**	2,343	62.0	**–**	**–**
I know how to adequately wash my hands/use hand sanitizer to prevent coronavirus^d^	2,804	73.8	**–**	**–**	2,423	64.2	**–**	**–**
I have the supplies I need to be able to frequently handwash/use hand sanitizer^d^	2,786	74.2	**–**	**–**	2,505	66.3	**–**	**–**
It is easy for me to frequently handwash/use hand sanitizer^d^	2,676	72.1	**–**	**–**	2,300	62.3	**–**	**–**
It takes a lot of effort for me to frequently handwash/use hand sanitizer (reversed) (individual item)	3,604	94.1	**–**	**–**	3,604	93.2	**–**	**–**
	**N**	**M**^e^	**SD**^f^	**Min, Max**	**N**	**M**^e^	**SD**^f^	**Min, Max**
Capability composite measure^d^	3,612	3.6	1.7	0, 5	3,608	3.2	1.9	0,5
**Opportunity**	**N**^**a**^	**%**^**b**^	**–**^**c**^	**–**^**c**^	**N**^**a**^	**%**^**b**^	**–**^**c**^	**–**^**c**^
Seeing handwashing signs in public reminds me to wash my hands/seeing hand sanitizer stations in public remind me to use hand sanitizer^g^	1,065	29.9	**–**	**–**	1,766	47.5	**–**	**–**
Seeing other people washing their hands/use hand sanitizer reminds me to wash my hands/clean my hands^g^	978	28.1	**–**	**–**	1,157	32.2	**–**	**–**
Frequent handwashing/hand sanitizer use is a habit in my everyday life	2,299	62.0	**–**	**–**	1,311	37.5	**–**	**–**
Lack of time can make it hard for me to frequently handwash/use hand sanitizer (reversed) (individual item)	3,479	95.2	**–**	**–**	3,521	96.7	**–**	**–**
	**N**	**M**^e^	**SD**^f^	**Min, Max**	**N**	**M**^e^	**SD**^f^	**Min, Max**
Opportunity composite measure^g^	3,612	0.6	0.8	0, 2	3,608	0.8	0.9	0, 2
**Motivation**	**N**^**a**^	**%**^**b**^	**–**^**c**^	**–**^**c**^	**N**^**a**^	**%**^**b**^	**–**^**c**^	**–**^**c**^
Frequent handwashing/use of hand sanitizer is a good way to protect myself from coronavirus^h^	2,865	76.4	**–**	**–**	2,207	59.3	**–**	**–**
Frequent handwashing/use of hand sanitizer will protect people I care about from coronavirus^h^	2,526	68.1	**–**	**–**	2,059	55.7	**–**	**–**
People I care about think frequent handwashing/use of hand sanitizer is important^h^	2,049	55.1	**–**	**–**	1,844	49.5	**–**	**–**
I could become sick with coronavirus if I do not handwash/use hand sanitizer frequently^h^	1,918	53.1	**–**	**–**	1,413	38.2	**–**	**–**
	**N**	**M**^d^	**SD**^e^	**Min, Max**	**N**	**M**^d^	**SD**^e^	**Min, Max**
Motivation composite measure^h^	3,612	2.5	1.5	0, 4	3,608	2.0	1.6	0, 4

^a^unweighted N

^b^ = weighted % agreement

^c^indicates that there are no standard deviation or minimum and maximum values because the item is dichotomous

^d^The Capability composite measure was made up of the first five individual capability items. For handwashing; the standardized alpha was 0.82, for use of hand sanitizer, the standardized alpha was 0.82. Higher scores on the composite variable indicate higher levels of agreement

^e^M = mean

^f^SD = standard deviation

^g^The Opportunity composite measure was made up of the first two individual Opportunity items. For handwashing, the standardized alpha was 0.74, for use of hand sanitizer, the standardized alpha was 0.70. Higher scores on the composite variable indicate higher levels of agreement

^h^The Motivation composite measure was made up of the four individual Motivation items. For handwashing, the standardized alpha was 0.79; for use of hand sanitizer, the standardized alpha was equal to 0.83. Higher scores on the composite variable indicate higher levels of agreement

We used results from exploratory factor analysis and Cronbach’s alpha to produce internally consistent, single factor COM-B constructs for the two hand hygiene behaviors. Based on the results of the analyses, we created a capability composite measure using five of the six capability items, an opportunity composite measure using two of the four opportunity items, and a motivation composite measure using all four motivation items (Table 2). Standardized Cronbach’s alpha are located in Table 2 and were as follows: Capability (Handwashing α = 0.82; Hand Sanitizer α = 0.82), Opportunity (Handwashing α = 0.74; Hand Sanitizer α = 0.70), Motivation (Handwashing α = 0.79; Hand Sanitizer α = 0.83). To create the composite measures, we summed the number of items respondents endorsed in each group of items. For example, if a respondent endorsed 2 of the 4 motivation items for handwashing, their score would be 2. The items that did not fit into the composite measures were treated individually. To aid interpretability, several items were reverse scored (Table 2).

### Other motivation variables

We also assessed other aspects of respondents’ motivation including perceived susceptibility to illness, perceived severity of illness, and perceived behavioral control. To assess these constructs, respondents were presented with a series of statements and asked if they agreed with them. One statement focused on perceived susceptibility (I am concerned about my own risk of infection with coronavirus), two focused on perceived severity (e.g., Getting sick with coronavirus can be life threatening), and three focused on perceived behavioral control (e.g., It is impossible to prevent coronavirus infection [reversed]). Exploratory factor analyses of the items indicated that these items should be treated individually. For interpretability’s sake, several items were reverse scored (Table 3).

**Table 3:** Percent agreement with statements assessing respondents’ perceived susceptibility to, severity of, and control over coronavirus infection – Fall Styles data, 2020 (*N* = 3,615^a^)

Survey item	N^a^	%^b^
**Perceived Susceptibility**		
I am concerned about my own risk of infection with coronavirus	1,910	49.2
**Perceived Severity**		
Getting sick with coronavirus can be life threatening	2,866	75.3
Most people who get coronavirus only experience mild symptoms (reversed)	2,306	65.6
**Perceived Behavioral Control**		
Whether or not I am infected with coronavirus, is beyond my control (reversed)	2,998	81.1
It is impossible to prevent coronavirus transmission (reversed)	3,083	84.8
If I am careful, I can avoid coronavirus infection	2,321	62.4

^a^unweighted N; *N* < 3,625 due to missing data

^b^weighted % agreement

### Analysis

We calculated weighted frequencies and means for the hand hygiene behaviors (Table 1), as well as measures of capability, opportunity, and motivation (Table 2). We then conducted a series of weighted multiple logistic regressions using backward variable selection based on Schwartz Bayesian criterion to create models for the hand hygiene behaviors. We used the capability, opportunity, and motivation measures as explanatory variables and controlled for six demographic variables often associated with hygiene behaviors (age, gender, race/ethnicity, education, income, region, Table 1) [7, 10, 11, 16]. Results are presented as adjusted odds ratios along with the 95% confidence intervals and probability values. All analyses were conducted using SAS 9.4. This activity was reviewed by the U.S. Centers for Disease Control and Prevention (CDC) and was conducted consistent with applicable federal law and CDC policy.^1^

## Results

### Descriptive analyses

#### Demographics

Respondent demographic characteristics, weighted to match the U.S. population, are included in Table 1. Respondents were primarily ≥ 60 years of age (29%), female (52%), and Non-Hispanic White (64%), had at least a Bachelor’s degree (34%), earned annually ≥ $100 k (38%), and lived in the South (38%).

#### Hand hygiene

Most respondents said they washed their hands often with soap and water (89%, unweighted *N* = 3,237; weighted *N* = 3,210) and were using alcohol-based hand sanitizer (72%, unweighted *N* = 2,584; weighted *N* = 2,591) to prevent coronavirus (Table 1).

#### Handwashing and COM-B

Respondents reported high levels of capability to engage in handwashing behavior, with almost all respondents reporting that it does not take a lot of effort to frequently wash hands (94%) and the majority reporting that they are capable of frequently washing their hands to prevent coronavirus (76%), have the supplies to wash hands frequently (74%), and know how to wash hands to prevent coronavirus (74%) (Table 2). About two-thirds (67%) of respondents reported knowing when they should wash their hands to prevent coronavirus. For opportunity variables, almost all respondents reported that lack of time did not make it hard to frequently handwash (95%) and 62% reported that frequent handwashing was a habit in their daily life. Fewer respondents indicated that seeing handwashing signs in public (30%) and seeing people washing their hands reminded them to wash their hands (28%). Finally, data on the motivation variables indicated that over three quarters of respondents believed that frequent handwashing was a good way to prevent illness with coronavirus (76%) and 68% perceived that frequent handwashing would protect people the respondent cared about from coronavirus. Just over half of respondents reported that people they care about think frequent handwashing is important (55%) or that they (the respondents) could become sick with coronavirus if they do not wash hands frequently (53%).

For the composite measures, respondents scored an average of 3.6 (out of 5; 0 = no items were endorsed, 5 = all five items endorsed) on the capability measure, an average of 0.6 (out of 2; 0 = no items were endorsed, 2 = both items endorsed) on the opportunity measure and an average of 2.5 (out of 4; 0 = no items were endorsed, 4 = all items endorsed) on the motivation measure.

#### Hand sanitizer use and COM-B

Respondents report high levels of capability to use hand sanitizer, with almost all respondents reporting that it does not take a lot of effort to frequently use hand sanitizer (93%) (Table 2). Two thirds of respondents reported that they have the supplies needed to frequently use hand sanitizer (66%), are capable of frequently using hand sanitizer to prevent coronavirus (65%), know how to adequately use hand sanitizer to prevent coronavirus (64%), that it is easy to frequently use hand sanitizer (62%) and know when to use hand sanitizer to prevent coronavirus (62%). With regards to opportunity, almost all respondents reported that lack of time did not make it hard to frequently use hand sanitizer (97%). Less than half of respondents, however, reported that seeing hand sanitizer stations reminded them to use hand sanitizer (48%), seeing other people use hand sanitizer reminded them to use hand sanitizer (32%), and that frequent hand sanitizer use is a habit in everyday life (38%). Finally, data from the motivation variables indicated that over half of respondents believed that frequent use of hand sanitizer was a good way to prevent illness with coronavirus (59%) and 56% perceived that frequent hand sanitizer use would protect people the respondent cared about from coronavirus. Less than half of respondents, however, reported that people they care about think frequent use of hand sanitizer is important (50%) or that they could become sick with coronavirus if they do not use hand sanitizer frequently (38%).

For the composite measures, respondents scored an average of 3.2 (out of 5; 0 = no items were endorsed, 5 = all five items endorsed) on the capability measure, an average of 0.80 (out of 2; 0 = no items were endorsed, 2 = both items endorsed) on the opportunity measure and an average of 2.0 (out of 4; 0 = no items were endorsed, 4 = both items endorsed) on the motivation measure.

#### Other motivation variables

Less than half (49%) of respondents said they were concerned about their own risk of infection with coronavirus (Table 3). Over 80% of respondents said that whether they were infected with coronavirus was within their control and that it was possible to prevent coronavirus. Over 60% of respondents agreed that if they were careful, they could avoid coronavirus infection. Seventy-five percent of respondents said that getting sick with coronavirus could be life threatening and 66% said that people who get coronavirus can experience severe symptoms.

### Multiple regression analyses

#### Handwashing

Multiple logistic regressions identified seven variables that were significantly associated (*p* < 0.05) with respondents reporting that they wash their hands often with soap and water to prevent coronavirus (Table 4). Those with higher perceived capability for handwashing had greater odds of self-reported frequent handwashing. Those who said frequent handwashing was a habit, which in this study was a measure of opportunity, more often reported frequent handwashing. On the other hand, those who said that public reminders to handwash reminded them to wash their hands, another measure of opportunity (the two items that make up the opportunity composite measure assess public reminders), less often reported frequent handwashing. Those with higher perceived motivation to wash their hands more often reported frequent handwashing. Those who perceived coronavirus to be a severe health problem (i.e., did not think that most people with coronavirus only experienced mild symptoms, thought getting sick with coronavirus could be life threatening) more often reported frequent handwashing. Finally, those who thought they could avoid coronavirus infection if they were careful more often reported frequent handwashing.

**Table 4:** Multiple regression adjusted odds ratios of measures of capability, opportunity, and motivation to wash hand often to prevent coronavirus infection – Fall Styles data, 2020 (*N* = 3,602)

	**Reduced Model**		
	**aOR**^**1**^	**95% CI**^**2**^	***p*-value**^**3**^
***Capability Variables***			
Capability Composite Measure	1.1	1.0, 1.2	.050
Capability individual item—It takes a lot of effort for me to frequently handwash	–	–	–
***Opportunity Variables***			
Opportunity Composite Measure	0.8	0.7, 1.0	.033
Opportunity individual item—Frequent handwashing is a habit in my everyday life	2.4	1.8, 3.1	< .001
Opportunity individual item—Lack of time can make it hard for me to frequently handwash (reversed)	–	–	–
***Motivation Variables***			
Motivation Composite Measure	1.8	1.6, 2.1	< .001
***Other Motivation Variables***			
Perceived susceptibility—I am concerned about my own risk of infection with coronavirus	–	–	–
Perceived severity—Most people who get coronavirus only experience mild symptoms (reversed)	1.5	1.2, 2.0	.002
Perceived severity—Getting sick with coronavirus can be life threatening	1.8	1.4, 2.4	< .001
Behavioral control—Whether or not I am infected with coronavirus, is beyond my control (reversed)	–	–	–
Behavioral control—It is impossible to prevent coronavirus transmission (reversed)	–	–	–
Behavioral control—If I am careful, I can avoid coronavirus infection	1.3	1.0, 1.7	.030

^1^OR = odds ratio; for the individual items, the OR is the odds that those who endorsed the item self-reported handwashing compared to the odds that those who did not endorse the item (reference level) self-reported handwashing. For the composite measures, the OR is the change in odds associated with each 1-unit increase in the measure

^2^CI = confidence interval

^3^*P* = probability value

^−−^ items included in the adjusted model that were not significantly associated with self-reported handwashing

#### Hand sanitizer use

Multiple logistic regressions identified six variables that were significantly associated (*p* < 0.05) with respondents reporting that they were using alcohol-based hand sanitizer to prevent coronavirus (Table 5). Those with higher perceived capability to frequently use hand sanitizer had greater odds of self-were more likely to reported hand sanitizer use to prevent coronavirus. Those who said frequent hand sanitizer use was a habit, a measure of opportunity, more often reported hand sanitizer use. Those with higher perceived motivation to frequently use hand sanitizer more often reported hand sanitizer use. Those who perceived themselves susceptible to coronavirus or who perceived coronavirus to be a severe health problem more often reported hand sanitizer use. Finally, those who thought that whether they became infected with coronavirus was within their control more often reported hand sanitizer use.

**Table 5:** Multiple regression odds ratios of measures of capability, opportunity, and motivation to use alcohol-based hand sanitizer to prevent coronavirus infection – Fall Styles data, 2020 (*N* = 3,600)

	**Reduced Model**		
	**aOR**^**1**^	**95% CI**^**2**^	***p*-value**^**3**^
***Capability Variables***			
Capability Composite Measure	1.6	1.5, 1.7	< .001
Capability individual item—It takes a lot of effort for me to frequently use hand sanitizer	**–**	**–**	**–**
***Opportunity Variables***			
Opportunity Composite Measure	**–**	**–**	**–**
Opportunity individual item – Frequently using hand sanitizer is a habit in my everyday life	2.5	2.0, 3.3	< .001
Opportunity individual item—Lack of time can make it hard for me to frequently use hand sanitizer (reversed)	**–**	**–**	**–**
***Motivation Variables***			
Motivation Composite Measure	1.3	1.2, 1.4	< .001
***Other Motivation Variables***			
Perceived susceptibility—I am concerned about my own risk of infection with coronavirus	1.6	1.3, 2.0	< .001
Perceived severity—Most people who get coronavirus only experience mild symptoms (reversed)	1.3	1.0, 1.5	.023
Perceived severity—Getting sick with coronavirus can be life threatening	**–**	**–**	**–**
Behavioral control—Whether or not I am infected with coronavirus, is beyond my control (reversed)	1.3	1.0, 1.6	.043
Behavioral control—It is impossible to prevent coronavirus transmission (reversed)	**–**	**–**	**–**
Behavioral control—If I am careful, I can avoid coronavirus infection	**–**	**–**	**–**

^1^OR = odds ratio; for the individual items, the OR is the odds that those who endorsed the item self-reported hand sanitizer use compared to the odds that those who did not endorse the item (reference level) self-reported hand sanitizer use. For the composite measures, the OR is the change in odds associated with each 1-unit increase in the measure

^2^CI = confidence interval

^3^P = probability value

^−−^ items included in the adjusted model that were not significantly associated with self-reported hand sanitizer use

## Discussion

These nationally representative data suggest that in the fall of 2020, most U.S. adults were washing their hands often and using hand sanitizer to mitigate against COVID-19 transmission. When compared to previously published estimates of handwashing (using the same survey methodology and same survey question), self-reported handwashing appeared to drop four percentage points (from 93 to 89%) between spring of 2020 and fall of 2020 [7] (there are no directly comparable figures for hand sanitizer use). Apparent decreases in handwashing over this time period were notable for non-Hispanic White people (from 94 to 88%), those with some college education (from 95 to 89%), and those in the Midwest (from 94 to 87%). Although it is not clear if these declines are statistically significant, decreases in self-reported hygiene behavior among U.S. adults has been reported elsewhere, with at least two studies reporting a decrease in handwashing behavior over the course of the pandemic [36, 37]. These decreases during the pandemic contrast to reported increases in self-reported handwashing behavior among U.S. adults from fall 2019 (before the COVID-19 pandemic) to summer 2020 (during the pandemic) [11]. As the current study examined cross-sectional data, it is not clear what caused the decrease in self-reported handwashing from Spring 2020 to Fall 2020. Perhaps the decrease is explained by the decrease in cases of COVID-19 in the U.S. during Summer 2020 [3]; this decrease in cases could have impacted the salience of health promotion messages and mitigation behaviors among U.S. adults. Future work should continue to monitor the public’s engagement in a range of COVID-19 mitigation behaviors and monitor factors that influence those behaviors. This work could provide more insight to explain shifts in self-reported behavior and could identify opportunities to promote recommended mitigation behaviors.

### Hand hygiene and capability

This study identified how components of the COM-B Model (capability, opportunity, motivation) were related to hand hygiene behaviors. The COM-B model states that individuals must have both the psychological and physical capability to engage in a specific behavior [32]. This includes knowledge and understanding of the behavior and physical skill to do the behavior. Although high proportions of respondents agreed with the capability statements concerning handwashing and hand sanitizer use, there is still room for improvement. For example, 24% of respondents reported they were not capable of washing hands frequently, and 35% reported they were not capable of using hand sanitizer. This study was not designed to assess factors that impact capability to engage in hand hygiene behaviors; future work should do so. Additionally, 33% and 38% of respondents did not know when they should wash their hands or use hand sanitizer, respectively, and 26% and 36% of respondents did not know how to adequately wash their hands or use hand sanitizer. Both of these behaviors are not novel, and U.S. adults were already engaging in them during the COVID-19 pandemic [7–15]. Additionally, there is evidence that levels of knowledge about the use of handwashing as a prevention strategy is high among U.S. adults [34, 38]. However, the present study’s results suggest that health communication efforts may need to not only provide information about the importance of hand hygiene behaviors, but also provide information about when to engage in these behaviors, and the steps needed to correctly engage in them.

The composite measure of capability was associated with both hand hygiene behaviors assessed in this study, although the relationship was stronger for hand sanitizer use than for handwashing. The finding of association between capability and hand hygiene is consistent with studies conducted both before and during the pandemic. In a pre-COVID-19 systematic review, White et al. (2020) found that knowledge of when to wash hands and having access to supplies were positively associated with handwashing [39]. A study using the COM-B model to identify predictors of a suite of hygiene behaviors during the COVID-19 pandemic also found a relationship between capability and engaging in hygiene behaviors [22]. These results suggest that efforts to promote hand hygiene during the COVID-19 pandemic among U.S. adults should consider addressing aspects of individual capability to engage in hand hygiene behaviors.

### Hand hygiene and opportunity

Opportunity in the COM-B model refers to the physical and social opportunities for individuals to engage in a specific behavior and includes factors such as having time or resources to do the behavior, cues to engage in the behavior, and interpersonal influencers such as social cues and norms [32]. Our data suggest that respondents had the physical opportunity of time to engage in hand hygiene; over 95% reported that lack of time did not make it hard to engage in frequent handwashing and hand sanitizer use. In contrast, however, most (60%) respondents said that visual cues (visual cues and seeing other people engage in hand hygiene) did *not* remind them to engage in hand hygiene behaviors. Indeed, the composite variable for opportunity, which included two items assessing visual cues, had a negative association with handwashing and was not associated with hand sanitizer use. This finding contrasts with work conducted before the COVID-19 pandemic that found that being cued by handwashing facilities tended to be positively associated with handwashing [39]. It is unclear why, in this study, visual cues were associated with respondents being less likely to report frequent handwashing behavior; additional studies are needed to further understand the relationship between cues and handwashing behaviors, including studies that assess frequency of observed handwashing behavior rather than self-reported behavior. Respondents’ perceptions of frequent handwashing and hand sanitizer use as habits in everyday life was the only opportunity measure associated with respondents’ self-reported handwashing and hand sanitizer use behaviors. The finding of positive associations between habits and hand hygiene is consistent with findings from other studies and suggests that supporting the formation of hand hygiene habits may be useful in efforts to promote hand hygiene during the COVID-19 pandemic (particularly so, given the relatively low percentages of respondents reporting hand hygiene habits) [40, 41]. Although habit was associated with self-reported hand hygiene behavior, only 38% of our respondents reported that frequent hand sanitizer use was a habit, and less than two-thirds reported that frequent handwashing was a habit in everyday life (62%). To promote hand hygiene behaviors, future communication approaches may want to focus on providing the public with strategies and tips to incorporate hand hygiene behaviors in daily routines. These strategies could include self-monitoring, planning, and behavior cues to foster habit formation [42].

Overall, however, our results suggest that when designing health communication, education, and behavior change interventions to promote hand hygiene behavior, a focus on the construct of opportunity may not be as useful as focusing on other constructs of the COM-B model.

### Hand hygiene and motivation

Of the three COM-B constructs evaluated in this study, motivation was most strongly associated with likelihood to report frequent handwashing and hand sanitizer use. The COM-B model posits that an individual must have enough motivation to engage in a particular behavior rather than engage in other behaviors or to not engage in any behaviors at all [32]. Our results support those of Miller et al. (2020) who also found that, overall, motivation was strongly associated with engaging in hygiene-related behaviors during the COVID-19 pandemic, with motivation more strongly associated with hygiene behavior than capability and opportunity [22].

Our data on motivation measures suggest that the majority of U.S. adults are motivated to engage in hand hygiene behaviors—they believe that frequent handwashing is a good way to protect themselves from coronavirus, and to a lesser extent feel this way about hand sanitizer. Perceived efficacy of recommended behaviors and outcome expectancies have been found to be associated with increased hand hygiene behavior during the COVID-19 pandemic [17, 26] and during previous pandemics [43], and with general determinants of hand hygiene behavior [39]. Of note, perceptions of effectiveness of mitigation behaviors have been found to be associated with knowledge about COVID-19 [44], suggesting that efforts to address this aspect of motivation should include a focus on increasing baseline knowledge of COVID-19.

Motivational measures of perceived susceptibility, severity, and behavioral control were associated with one or both of the hand hygiene behaviors. These findings support other research showing significant relationships between these constructs and hand hygiene in the COVID-19 pandemic [8, 10, 23, 26], during other pandemics [43], and in general [39]. Data collected during the COVID-19 pandemic shows significant variability in individuals’ perceptions of susceptibility and severity [8, 10, 23, 26]. Given the multiple factors that influence susceptibility to and severity of COVID-19, particularly before the availability of vaccines, when these data were collected, this variability is perhaps not surprising. However, it does indicate that future efforts to increase prevention behaviors may need to focus on clear communication concerning these concepts. Researchers may also wish to explore factors that influence perceived susceptibility to and severity of COVID-19. Indeed, it would likely be useful to identify factors that influence all the constructs of the COM-B model.

This study is subject to several limitations. First, although the data were weighted to be nationally representative and computers and internet access were provided to respondents who needed them, it is possible that results may not be fully representative of the U.S. adult population (e.g., those uncomfortable with the internet may have been less likely to participate). Second, the items developed for this study were not piloted prior to use. Correspondingly, composite measures were created post hoc and may lack construct validity. Third, the data might be affected by social desirability bias, which might have resulted in overreporting of agreement with statements assessing COM-B constructs and of socially desirable behaviors, such as hand hygiene behaviors. Indeed, self-reported handwashing rates are consistently higher than observed handwashing rates [45]. Finally, data for this study were cross-sectional and as such we cannot make causal statements about the directionality of the relationships between hand hygiene behaviors and COM-B constructs.

## Conclusion

The COM-B model was a helpful framework for increasing our understanding of hand hygiene behavior; it identified capability and motivation, and to a lesser extent, opportunity, as predictors of both handwashing and hand sanitizer use during the COVID-19 pandemic, prior to the availability of vaccines. We also found that the motivational measures of perceived severity and susceptibility to illness and behavioral control were positively associated with handwashing and hand sanitizer use. These findings highlight the need for health promotion strategies to focus on increasing and maintaining capability, opportunity, and motivation to engage in the targeted hand hygiene behaviors. The Behavior Change Wheel [32] outlines approaches that can be used to target these constructs in various behavior change interventions. This includes strategies such as increasing capability through improving knowledge and understanding and increasing opportunity through habit formation. These findings can inform efforts to increase hand hygiene during the COVID-19 pandemic and other hand hygiene promotion efforts.

## Data Availability

The data that support the findings of this study are available from Porter Novelli Public Services but restrictions apply to the availability of these data, which were used under license for the current study, and so are not publicly available. Data are however available from the authors upon reasonable request (email Laura Brown at lrg0@cdc.gov) and with permission of Porter Novelli Public Services (deanne.weber@porternovelli.com).

## Footnotes

1 45 C.F.R. part 46, 21 C.F.R. part 56; 42 U.S.C. Section 241(d); 5 U.S.C. Section 552a; 44 U.S.C. Section 3501 et seq.
